# The Pyruvate–Glyoxalate Pathway as a Toxicity Assessment Tool of Xenobiotics: Lessons from Prebiotic Chemistry

**DOI:** 10.3390/jox15060198

**Published:** 2025-12-01

**Authors:** François Gagné, Chantale André

**Affiliations:** Aquatic Contaminants Research Division, Environment and Climate Change Canada, Montréal, QC H2Y 2E7, Canada

**Keywords:** prebiotic chemistry, pyruvate–glyoxalate pathway, biotest, alternative toxicity test

## Abstract

There is an urgent need to evaluate the toxicity of xenobiotics and environmental mixtures for preventing loss in water quality for the sustainability of aquatic ecosystems. A simple prebiotic chemical pathway based on malate formation from pyruvate (pyr) and glyoxalate (glyox) is proposed as a quick and cheap screening tool for toxicity assessment. The assay is based on the pyr and glyox (aldol) condensation reactions, leading to biologically relevant precursors such as oxaloacetate and malate. Incubation of pyr and glyox at 40–70 °C in the presence of reduced iron Fe(II) led to malate formation following the first 3 h of incubation. The addition of various xenobiotics/contaminants (silver, copper, zinc, cerium IV, samarium III, dibutylphthalate, 1,3-diphenylguanidine, carbon-walled nanotube, nanoFe_2_O_3_ and polystyrene nanoparticles) led to inhibitions in malate synthesis at various degrees. Based on the concentration inhibiting malate concentrations by 20% (IC20), the following potencies were observed: silver < copper ~ 1.3-diphenylguanidine ~ carbon-walled nanotube < zinc ~ samarium < dibutylphthalate ~ samarium < Ce(IV) < nFeO_3_ < polystyrene nanoplastics. The IC20 values were also significantly correlated with the reported trout acute lethality data, suggesting its potential as an alternative toxicity test. The pyr-glyox pathway was also tested on surface water extracts (C18), identifying the most contaminated sites from large cities and municipal wastewater effluents dispersion plume. The inhibition potencies of the selected test compounds revealed that not only pro-oxidants but also chemicals hindering enolate formation, nucleophilic attack of carbonyls and dehydration involved in aldol-condensation reactions were associated with toxicity. The pyr-glyox pathway is based on prebiotic chemical reactions during the emergence of life and represents a unique tool for identifying toxic compounds individually and in complex mixtures.

## 1. Introduction

Aquatic ecosystems are subjected to multiple sources of pollution, releasing thousands of human-made xenobiotics through water (wastewaters and rainfall runoffs), air (dust, volatile compounds) and solids (solid waste, electronic waste, etc.). It is generally accepted by the scientific community that relying only on the chemical composition of water/sediments/air provides incomplete information and that toxicity assessments are required, which integrate the whole-mixture effects and unknown chemicals [1]. The ongoing toxicity evaluations of these complex mixtures are nowadays an integral part of ecotoxicological risk assessment strategies. In the context of environmental protection strategies and law enforcement, bioassays investigating the toxicological properties of these chemicals and mixtures represent an ongoing effort for ensuring sustainability of our economy. Bioassays usually encompass the selection of sentinel species at specific levels of the food chain where fish toxicity information is mandatory since they are protected and recognized by law [2]. For example, a test battery encompassing bacteria, algae/plants, invertebrates (worm, crustacean and bivalve) and vertebrates (fish and amphibians) helps identify the most sensitive species [3] and elaborate a toxicity distribution profile often used for probabilistic risk assessments of chemicals of emerging interest [4]. Fish toxicity testing for regulatory purposes leads to the sacrifice of tens of thousands of fish per country. In this context, alternative methods for limiting fish use are welcome for ethical and practical consideration. Different types of alternatives exist, albeit not systematically validated: fish at the hatching/larval stage, alternate organisms (usually an invertebrate), in vitro (cells) methods, biosensors (enzymes), biochemical pathways and artificial intelligence modeling in identifying potentially toxic chemicals based on various chemical properties. For example, the hydra *Hydra vulgaris* bioassay and the peroxidase biosensor hold promise as alternatives given their high sensitivity and predictability in the rainbow trout acute lethality test for law enforcement [5,6]. Metabolic pathways have yet to be considered as alternative tests despite their potential to deliver highly cost-effective biotests. Metabolic pathways essentially determine whether xenobiotics could dampen parts of crucial metabolic networks such as the tricarboxylic acid (TCA) cycle for energy metabolism and cellular respiration involving the five universal intermediates—acetate, pyruvate, oxaloacetate (malate), succinate and α-ketoglutarate—found in all life forms [7].

**Figure 1:**
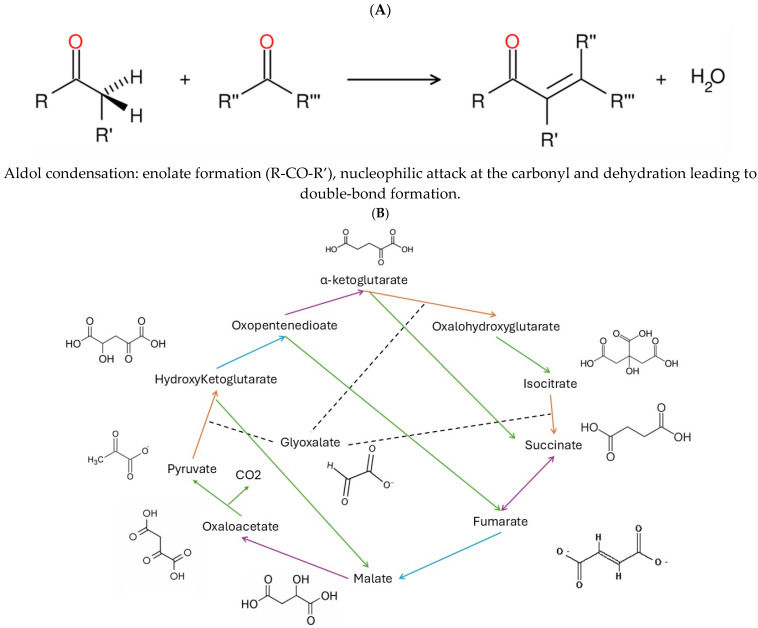
The pyruvate–glyoxalate pathway. The pyr-glyox pathway starts with the aldol reaction depicted in (**A**). The pathway between pyruvate and glyoxalate in the presence of reduced Fe^2+^ (**B**). The cycle involves aldol/retro-aldol condensation (orange arrows, e.g., pyr + glyox → hydroxyglutarate and isocitrate → succinate + glyoxalate for aldol and retro-aldol reactions, respectively), hydration/retrohydration (blue arrows, e.g., hydroxyglutarate → oxopentenedioate), oxidative decarboxylation (green arrows, oxalohydroxyglutarate → isocitrate + CO_2_) and reduction/oxidation (violet arrows, e.g., succinate → fumarate). From [9].

In the selection of metabolic pathways as potential screening tools, primitive metabolic pathways thought to originate on early Earth before the formation of organized cells could be ideal candidates. These pathways intervened during the first prebiotic chemical reactions during the Hadean era of Earth [8]. These protometabolic networks could serve as toxicity alternatives provided that the generated toxicity data predicts toxicity in higher life forms. The TCA cycle has been an attractive target given that virtually all anabolic intermediates in the metabolic pathway chart originate from the TCA cycle found at the centre of metabolic pathways charts [9]. Evolutionary genomic studies suggest that an ancestor of the TCA cycle was formed in the making of intermediates such as sugars, amino acids and lipids. Called the reverse TCA (rTCA), it was autocatalytic before the formation of enzymes provided that the intermediates were maintained in the cycle (microenvironment), leading to the mobilization of carbon from the reduction of CO_2_ (abundant on early Earth). Indeed, the capacity of iron to promote the rTCA variant from simple prebiotic precursors such aspyruvate (pyr) and glyoxalate (glyox)—was experimentally established [10]. In addition, pyr and glyox could be produced from abiotic processes of atmospheric CO_2_ and N fixation by reduced iron [11]. Reduced iron Fe(II) was/is one of the most abundant metals in Earth’s crust, and large amounts of Fe(II) prior to the rise of oxygen were readily available in these times [12]. The reaction is surprisingly simple, requiring only heating of a mixture of pyr, glyox and Fe(II) under inert conditions (N2 atmosphere) for a few hours. This leads to the formation of highly interconnected pathways capable of both anabolic and catabolic activities involving five main reaction mechanisms: decarboxylation, reduction/oxidation, dehydration/hydration and aldol/retro-aldol reactions (Figure 1). Aldol condensation, in turn, involves the formation of enolate ions (pyr), nucleophilic attack on the carbonyl/aldehyde carbon and dehydration (Figure 1). It is therefore theorized that xenobiotics hindering these prebiotic reactions have the potential to initiate toxicity.

The purpose of this study was therefore to examine the pyr-glyox pathway to probe the effects of selected xenobiotics and mixtures (surface wastewater extracts) in the formation of the end-product malate. The hypothesis states that xenobiotics capable of hindering malate formation from the primitive pyr-glyox pathway equates to toxicity. The influence of 10 well-known contaminants of emerging interest (plasticizers, endocrine disruptors, plastic/carbon/iron-based nanoparticles, tire wear substances, metals and rare earth elements) on the inhibition of the pyr-glyox pathway was examined and compared to fish mortality data. Moreover, malate production was examined in real-life river water and wastewater extracts (mixtures) to determine the most impacted/polluted samples. An attempt was made to understand the role of ancient metabolic pathways beyond the oxidative stress paradigm during the initiation of toxicity [13].

## 2. Materials and Methods

### 2.1. Chemicals and Water Extraction

Chemicals at 99% purity, copper (CuCl_2_), silver (AgNO_3_), samarium (SmCl_3_), cerium (CeCl_4_), zinc (ZnSO_4_), the plasticizer/endocrine disruptor dibutylphthalate (DBP) and tire-related compound 1,3-diphenylguanidine (1,3-DPG), were purchased from Sigma Chemical Company (Oakville, ON, Canada). They were prepared in MilliQ water at 1 mg/mL with the exception of DBP and 1,3-DPG, which were prepared in ethanol at the same concentration. Polystyrene nanoparticles of 20 nm diameter were purchased from ThermoFischer Scientific (Waltham, MA, USA). They were diluted in MilliQ water at 0.1%. Carbon nanotubes (CNTs) of 5–10 nm internal diameter/1–3 µm fiber length and iron (II) nanoparticles (nFe(II)_2_O_3_, 30 nm diameter) were purchased at US Research Nanomaterials (Houston, TX, USA). They were diluted at 10 mg/mL in MilliQ water to prevent aggregation [14]. The stock solutions were prepared the day before the exposure experiments. All concentrations are nominal concentrations.

Surface waters, untreated wastewaters (influent) and treated wastewaters (effluent) were collected as composites of surface waters (3 × 1 L samples per site) and 24 h composites (1 L) for influents and municipal wastewaters, respectively. The surface waters were collected in Saint-Lawrence in the Montreal area: at the mouth of the Chateaughay river (CHA) on the south shore of the island of Montreal, at one site 8 km downstream of the city of Montréal (Downs) and at the marina of the city of Lavaltrie (LA) located some 40 km downstream of the island of Montreal on the north shore of the Saint-Lawrence river. The untreated (influent) and treated wastewaters (effluent) were collected from a typical city with a population of circa 100,000 residents using a primary clarifier (ferric chloride to remove phosphates) followed by an activated sludge and chlorine disinfection (sodium sulfite for dichlorination) treatments. A volume of 200 mL of sample was filtered on a 0.8 µm cellulose acetate membrane for the removal suspended solids, and the filtrate was passed through a C18 solid-phase extraction cartridge (Bond Elut, 100 mg bed mass, Santa Clara, CA, USA), washed with 5 mL of MilliQ water and eluted with 0.5 mL ethanol corresponding to 400× concentrates. The ethanol extracts were kept at 4 °C until analysis the same week.

### 2.2. Pyruvate–Glyoxylate Assay

The pyruvate–glyoxylate assay was prepared following a modification of a previously reported methodology [9,10]. The main modifications were the reduction of reactant concentrations to increase sensitivity to exposure to various chemicals and complex mixtures in the environment and the optimal detection of the end-products malate and oxaloacetate using an enzyme detection system. Briefly, 1 mM of sodium pyruvate, 2 mM glyoxalate and 4 mM FeSO_4_ were prepared in 1 mL of MilliQ water at pH 6.5 with increasing concentrations (in separate tubes: control, 4, 20 and 100 mg/L) for each of the 10 contaminants highlighted above or water extracts (in separate tubes: control, 0.8, 4 and 20X). The assay tubes were prepared in duplicate samples and the experiment repeated 3 times. The mixture was degassed under a N_2_ stream and incubated at 70 °C for 3h. The controls contained only MilliQ water or the equivalent amount of ethanol (C18 extract solvent; final concentration of 5% for the 20X concentration). The reaction tubes appeared dark yellow from Fe(II), and there was no observation of turbidity. The reaction was cooled down at room temperature, and the levels of malate were determined by the malate dehydrogenase enzyme system. A 10–20 µL sample of the reaction mixture was mixed with 1 mM NAD^+^ in the assay buffer composed of 140 mM NaCl, 1 mM MgCl_2_ and 20 mM Tris base, pH 8.5, in 96-well dark microplates. The reaction was initiated by adding 10 µL of 10 units of malate dehydrogenase (10 units of MDH; Sigma Chemical Company, Oakville, ON, Canada). An operational blank was composed of NAD^+^ alone. Malate standards (0.05–0.15 mM) in the presence of NAD^+^ and MDH were also included for calibration. The formation of reduced NADH was measured for 20 min at 1 min intervals by fluorescence at 350 nm excitation and 450 nm emission wavelengths using a microplate reader (Synergy IV, Biotek instruments, Winooski, VT, USA). The rate of NADH formation was calculated for 10 min and used to determine the formation of malate in duplicate assay samples. The data is expressed as relative fluorescent unit (RFU)/min.

### 2.3. Rainbow Trout Acute Lethality Tests

The acute toxicity data of rainbow trout (*Oncorhynchus mykiss*) or other fish species (*Channa punctatus*, *Oryzias latipe* and *Cyprinus carpio*) were provided in the literature for the tested compounds (with the exception of PSNPs) for comparison of malate inhibition with toxic effects on trout survival. With the exception of PsNPs, the acute lethality tests were performed following the standard protocol of Environment and Climate Change Canada [15] (biological test method: acute lethality test using rainbow trout—Canada.ca). Briefly, 10 newly hatched juveniles with the egg yolk sac fully reabsorbed (0.3–2.5 g) were placed in 60 L containers lined with polyethylene bags and exposed to increasing concentrations of PsNPs (100, 50, 10, 2 and 0.4 mg/L) for 96 h at 15 °C under constant aeration. The fish were not fed during the exposure period, and distressed fish were examined twice daily and immediately euthanized in 50 mg/L tricaine sulfate as per the animal care committee recommendations. A positive control (ZnSO_4_; LC50 0.4 mg/L (0.09–1.95% confidence interval)) was also used to ensure reproducibility of the fish toxicity test.

### 2.4. Data Analysis

The experiments were repeated *N* = 3 times, and the data were subjected to an analysis of variance after checking for data homogeneity using Levene’s and Shapiro–Wilk’s tests. In the case of non-parametric data, the data were log-transformed. Critical differences between controls and exposure concentrations were determined by the Least Square Difference test. The calculation of the concentration of the test samples that inhibited malate formation by 20% (IC20) was based on graphical extrapolation of the concentration–response curves. The lethal concentration that caused trout mortality by 50% (LC50) was calculated by the Spearman–Karber methodology [16]. The data are expressed in mg/L for single substances and X-fold concentration for complex mixtures (C18 ethanol extracts), where 1× concentration corresponds to the original undiluted water sample.

## 3. Results and Discussion

**Figure 2:**
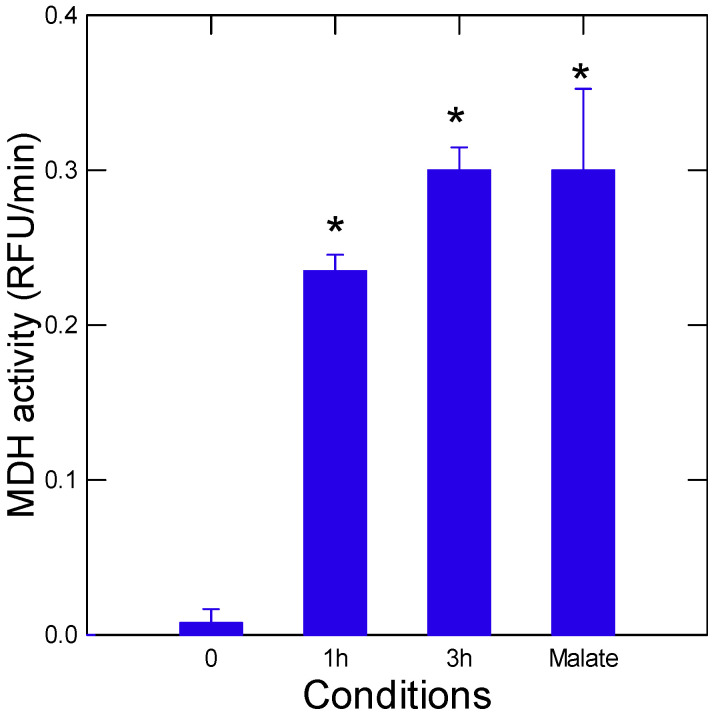
Formation of malate following incubation of pyruvate and glyoxalate. Pyr, glyox and reduced iron (Fe^2+^) were allowed to react at 70 °C for 1 and 3 h. Malate levels were determined by the NAD^+^ malate dehydrogenase system. A gradual increase in malate is observed relative to initial time 0 h at 1 and 3 h incubation times. The last column represents a malate standard (0.5 µM) added as a positive control for the malate detection aassay. The star symbol indicates significance at *p* < 0.05.

The pyr-glyox reaction involves a series of aldol-condensation, redox and dehydration reactions as depicted in Figure 1A. The newly formed metabolite hydroxyglutarate from glyox and pyr undergoes a series of hydration/dehydration, oxydo/reductive and additional aldol-condensation reactions, forming more complex molecules such as citrate, succinate, malate and oxaloacetate (Figure 1B). The source of electrons is assured by Fe(II), which represents one of the most abundant and available metals in Earth’s crust prior to the Grand Oxidation event [12]. When glyox and pyr were added at a molar ratio of 2:1 in the presence of 4 equivalents of reduced Fe(II) in MilliQ water, malate (and oxaloacetate) was detected as early of 1 to 3 h incubation at 70 °C (Figure 2). Longer exposure times up to 24 h led to increased levels of malate as well using the original protocol, but malate levels did not increase after 4 h when using lower concentrations of pyr, glyox and Fe(II). Indeed, the initial protocols used concentrations in the 0.1 to 0.4 M range, representing concentration levels much higher than environmental contaminants. The concentration was downscaled to the mM range, which improved the sensitivity of the assay toward environmental pollutants and permitting malate detection using the malate dehydrogenase assay. In these conditions, longer exposure times (24 h) at 70 °C did not lead to a significant increase in malate, probably due to saturation of the reaction or heat degradation. Hence, for toxicity experiments, the following concentrations of the reagents were used: 1 mM Pyr, 2 mM glyoxalate and 4 mM Fe(II) at 70 °C for 3h.

**Figure 3:**
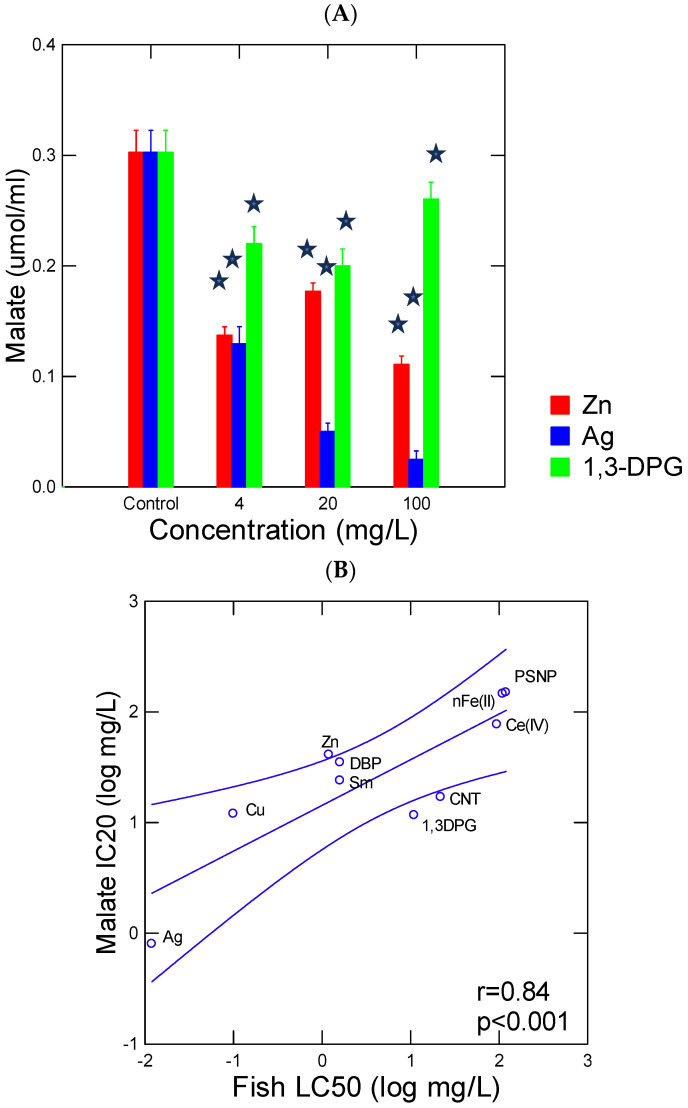
Representation of responses of malate formation inhibitions by selected toxic compounds toward rainbow trout. Representative inhibition profiles of malate production (**A**) and regression analysis of malate inhibition (log IC20 mg/L) and 96 h trout mortality (log LC50 mg/L) (**B**) from reported literature data. The regression slope between the IC20 of the pyr-glyox reaction and trout 96 h LC50 was obtained from *N* = 10 compounds reported in Table 1: DBP, 1,3-DPG, Cu, Ag, Zn, Sm, Ce(IV), PSNPs, CWNT, and nFe(II)_3_O_4_. The star symbol indicates significance at *p* < 0.05.

The pyr-glyox pathway was examined with increasing concentrations of the selected environmental contaminants (Figure 3A). The figure shows that increasing the concentrations of Ag, Zn and 1,3-DPG decreased production of malate at various degrees of intensity. Not only the pro-oxidant compound Ag^+^ but also zinc and 1,3-DPG (a tire-related compound) decreased malate production. This tire wear compound (1,3-DPG) was less potent than Zn in the inhibition ofmalate production. This suggests that not only oxidants but also other chemicals (reductants) can inhibit the pyr-glyox pathway. It is generally thought that the oxidative properties of compounds are the main drivers of toxicity of xenobiotics [13]. The concentration that inhibits malate concentration by 20% (IC) was calculated for the selected 10 xenobiotics and isreported in Table 1. The IC20 spans from 1 to 150 mg/L with the following decreases in potency to inhibit the pyr-glyox reaction: Ag > Cu ~ 1.3-DPG ~ carbon-walled nanotube > Zn > DBP > Sm > Ce(IV) > nFeO_3_ > polystyrene nanoplastics. With respect to fish toxicity, the LC50 ranged from 0.1 to 100 mg/L in the following decreasing order of toxicity: Ag > Cu > Zn ~ Sm ~ DBP > 1.3-DPG > carbon-walled nanotubes > nFe_2_O_3_ > polystyrene nanoplastics. Based on these distributions, it appears that the pyr-glyox pathway is as sensitive to oxidants as fish based on copper and Ag responses, albeit at concentrations 10 times higher than for rainbow trout. This could be attributed to the initial concentration of pyr (87 mg/L) and glyox (140 mg/L) used here where the initial concentreations could be reduced further as permitted by the malate detection system in place. The pyr-glyox reaction seems to respond more to electron donors (reductants) than rainbow trout, such as 1,3-DPG and carbon (graphene)-walled nanotubes in the presence of reduced iron Fe(II). Aldol condensation involves three major steps, including the formation of the enolate ion, where the mobility of hydrogen at the α carbon (of an C^δ−^–H^δ+^) next to carbonyl (pyruvate) could be inhibited by xenobiotics such as guanidines or acidic groups (carboxylic acid, thiols) in alkaline conditions [17]. Hence, this encompasses a large range of polar compounds such as pesticides, pharmaceuticals and industrial pollutants. For example, guanidines are used in the production of plastics (plastic tubing in households) and tire rubbers such as 1.3-DPG and 2-cyanoguanidine [18]. They are also considered chaotropic agents able to denature proteins and nucleic acids [19,20]. The former study also showed that excess oxidized Fe(II), cadmium, lead, manganese and aluminum were also able to inhibit this reaction, perhaps at the second step involving the nucleophilic reaction of the enolate ion. The guanidine-containing drug metformin was shown to inhibit the aconitase reaction involving the dehydration of isocitrate to citrate in the tricarboxylic acid cycle, suggesting interactions at the third step of aldol condensation as well [21]. Acrylamide and nucleophile maleimide were also shown to disrupt the cyclic production of thiol from amino acid thioester conjugates, which also involve nucleophilic reactions with thiols as with aldol reactions [22]. This suggests that electron donors/nucleophiles (RSH, maleimides, amines, etc.) could inhibit the pyr-glyox pathway but at different steps of the aldol-condensation reactions. The aldol condensation of 2-keto-4-hydroxybutyrate from glyox and pyr catalyzed by aldolase in bovine liver and *Escherichia coli* also revealed inhibitions by various agents [23]. It was found that halides (anions) and carboxylic acids were inhibitors while esters were less potent inhibitors of the condensation reaction of glyox and pyr. Mono-, di- and tricarboxylic acids were proportionally inhibitory, and hydroxypyruvate (blocks the mobility of H^+^ at the α carbon and the formation of enolate ion) was the most potent inhibitor. Increasing the concentration of glyox relative to pyr was also inhibitory, perhaps through the enzyme inactivation by this aldehyde. Hence, the pyr-glyox reaction involves reactions beyond the classic oxidative paradigm of toxic compounds. Indeed, compounds able to disrupt the formation of enols from the mobility of hydrogen of α carbon adjacent to carbonyl, and block/compete with the nucleophilic reaction to aldehydes and dehydration steps could initiate toxicity at the molecular level.

**Figure 4:**
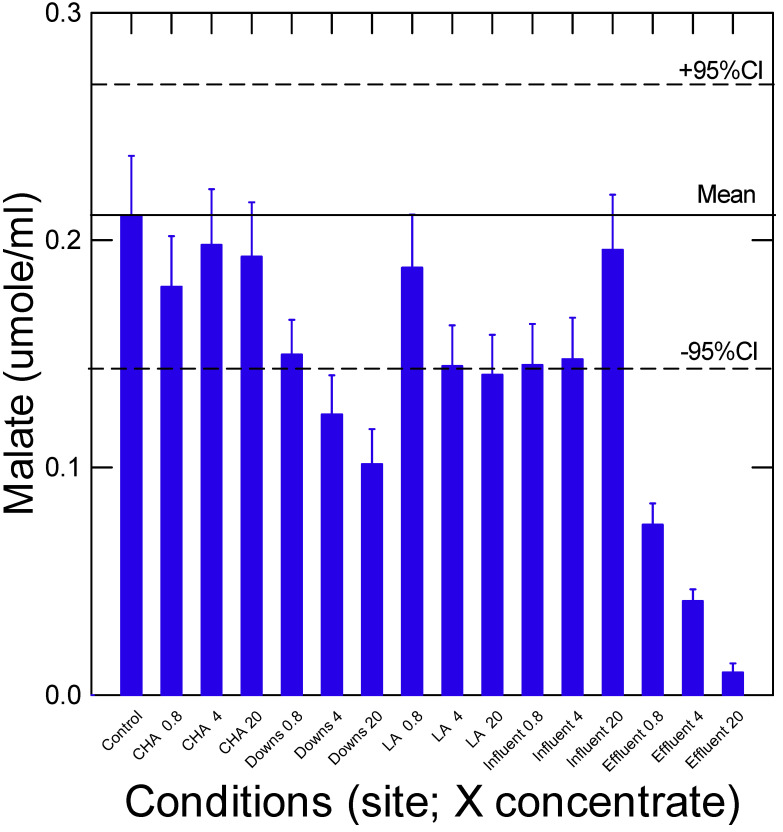
Influence of surface waters and wastewaters on the pyr-glyox pathway. Surface water and municipal influent/effluent extracts were examined with the pyr-glyox assay in triplicates (*N* = 3). The identification of the tested water samples and exposure concentration (expressed as X-fold concentration) are identified on the abscissa axis.

In an attempt to provide a toxicological meaning regarding this prebiotic reaction network, the malate IC20 was compared with the rainbow trout acute lethality tests (Figure 3B). The data show that concentrations able to reduce malate production were significantly correlated with trout toxicity (LC50) data (r = 0.84; *p* < 0.001). The LC50 value of PSNP was set at 100 mg/L (logLC50 = 2) since it was reported that this plastic induces oxidative stress and damage at this concentration [27,31]. This suggests that compounds able to inhibit aldol-condensation and/or redox properties are involved in the toxicity of these substances. These reactions take place in the TCA cycles present today, where inhibitions in these reactions will decrease energy production and cellular respiration rates. This could form the basis of toxicity initiation in organisms. The oxidative properties of xenobiotics represent one of the most fundamental interactions in toxicity initiation [13,32]. From the 10 selected compounds, 3 were nanoparticles (nFe_2_O_3_, PSNPs and CNT) and were shown to reduce malate production at relatively higher concentrations (20–100 mg/L), in keeping with their relatively low toxicity (compared to Ag or Cu) in fish and hydra in previous studies [26,33]. However, plastic nanoparticles and CNT produce oxidative stress in the mg/L range in hydra [30,31], suggesting impacts at longer exposure times. In this respect, the pyr-glyox pathway could serve as a generic test for toxicity screening of miscellaneous chemicals and mixtures. Keeping this in mind, we used the pyr-glyox reaction on C18 ethanolic extracts of various surface waters and wastewaters (Figure 4). The samples consisted of surface waters such as river water (CHA, LA, Downs) and untreated (influents) and treated effluents. The data revealed that river water samples did not produce significant changes in the reaction at concentrations reaching 20× of the water sample. The site downstream (DOWNS, 10 km downstream) of a 1.8-million-population city inhibited the pyr-glyox pathway at 20× concentration and to some extent at 4× but not at an environmentally realistic concentration, since no significant changes occurred at <1× (0.8) concentration. While the influent did not produce any significant changes in malate levels, the treated municipal effluent significantly inhibited malate formation at concentrations of 0.8× and higher. This suggests that this effluent treatment might pose a risk to aquatic life near the discharge point. In order to preserve anonymity of the city involved, fish toxicity of this effluent was previously reported on some occasions. This effluent is produced from an activated sludge effluent with residency times of 10–20 days, where ferric chloride for phosphate removal and disinfection (UV) steps was applied. Activated sludges of a city of similar size revealed substantial amounts of plastic (polystyrene) polymers (10–20 µg/L) and polyaromatic hydrocarbons (30–40 µg/L) in effluents [33]. The levels of melamine and polystyrene nanoplastics in the effluents were higher than in the influent, suggesting that sustained effluent treatment breaks down the suspended organic matter, leading to the release of dissolved components such as melamine-based products (paints and panels) and plastic nanoparticles [34]. This could explain why stronger inhibitions were observed in these secondary treated aeration sludge effluents than in the other samples. Moreover, 1,3-DPG (0.04 µg/L) and cyanoguanidine (2.4 µg/L), constituents of tire and tire wear dusts, are finding their way into the dissolved fraction of municipal effluents [35].

In conclusion, a rapid and cost-effective chemical test is proposed as a screening tool for miscellaneous environmental liquids. The chemical is based on biochemical reactions between biogenic precursors pyr and glyox as primitive biochemical pathways thought to originate on early Earth. The inhibition potencies of the selected test compounds revealed that not only pro-oxidants are associated with toxicity but chemicals hindering enolate formation, nucleophilic attack of carbonyls and dehydration. Furthermore, comparative analysis with fish toxicity (LC50) of pyr-glyox inhibitions revealed a significant correlation for the 10 compounds tested here, making this test a useful screening test for identifying potential toxic compounds. However, future research is necessary to validate this relationship, and the test should be further investigated with other toxic xenobiotics.

## Figures and Tables

**Table 1:** Selected compounds for the pyr-glyox reactions.

Compounds	Pyr-Glyox (IC20 mg/L)	Trout Toxicity (LC50 mg/L)	References
Dibutylphthalate (DBP)	35	1.6	[24]
1,3-Diphenylguanidine (1.3-DPG)	12	4.2	[25]
Copper Cu(II)	12	0.1	[26]
Silver Ag(I)	1	0.02	[26]
Zinc Zn(II)	41	1.6	[26,27]
Samarium Sm(III)	24	2	[28]
Cerium Ce(IV)	77	95	[28]
nFe_2_O_3_	146	100 (*Oryzias latipe* embryo)	[29]
Polystyrene nanoplastic (PSNP)	150	>100	This work (Section 2.3)
Carbon-walled nanotubes (CNTs)	17	22 (*Channa punctatus* juvenile)	[30]

## Data Availability

The original contributions presented in this study are included in the article/Supplementary Material. Further inquiries can be directed to the corresponding author.

## Supplementary Materials

The following supporting information can be downloaded at: https://www.mdpi.com/article/10.3390/jox15060198/s1, File S1: 621.2-CMP Elements Critiques pour Tech-2024-25.
