# Supplementary material for: The Pyruvate–Glyoxalate Pathway as a Toxicity Assessment Tool of Xenobiotics: Lessons from Prebiotic Chemistry
Source: J Xenobiot. 2025 Dec 1;15(6):198. doi: 10.3390/jox15060198 (PMC12734312; doi:10.3390/jox15060198)
Supplement: Supplementary file 1 [file jox-15-00198-s001.zip › jox-3924131-supplementary.pdf]

Laboratoire des essais environnementaux du Québec
105, rue McGill
Montréal, QC
H2Y 2E7

Environnement et Changement climatique Canada | Environment and Climate Change Canada

Certificat d'analyse

621.2-CMP Elements Critiques pour Tech-2024-25

François Gagné
Sciences et technologie, Eau
Recherche sur les contaminants aquatiques

105 rue McGill, 7e étage
Montréal, QC
H2Y 2E7

Demande d'analyse: Q25A046

Émis le: 2025-06-05
Imprimé le: 2025-06-05

Autorisation:

Geneviève Farley pour Serge Moore
Gestionnaire LEEQ

Les résultats émis via le certificat d'analyse portent uniquement sur les échantillons tels que reçus et analysés au laboratoire d'Environnement et Changement climatique Canada (ECCC) indiqué au certificat. Afin de s'assurer que des parties de ce certificat d'analyse ne soient prises hors contexte, ce certificat ne doit pas être reproduit, sinon en entier, sans l'autorisation du laboratoire.

Les laboratoires d'ECCC sont accrédités par la Canadian Association for Laboratory Accreditation (CALA) selon la norme ISO/IEC 17025 pour chacun des analytes rapportés, à l'exception de ceux comportant un astérisque (*). Veuillez consulter le site Web de la CALA (www.cala.ca) pour voir toute la portée de l'accréditation.

Canada

REMARQUES GÉNÉRALES

Unités	Description
%(v/v)	Pourcentage (volume/volume)
mg/L	milligramme par litre

Note	Description
*	Analyse/Analyte non accrédité(e)
ND	Non détecté à la limite de détection (LDM)
NR	Non récupéré

DESCRIPTION DE L'(DES) ÉCHANTILLON(S)

No Labo	Nom éch.	Station	Matrice	Date/heure de prélèvement	Date de réception	Type d'échantillon
**Q25A046-01**	*6PPD*		Eau	2025-01-30 08:00 EST<br>Échantillonné par: Eva Roubeau	2025-01-30	Instantané
**Q25A046-02**	*Mix Leachate*		Eau	2025-01-31 08:00 EST<br>Échantillonné par: Eva Roubeau	2025-01-30	Instantané
**Q25A046-03**	*6PPDQ*		Eau	2025-02-06 08:00 EST<br>Échantillonné par: Eva Roubeau	2025-01-30	Instantané
**Q25A046-04**	*HMMM*		Eau	2025-02-07 08:00 EST<br>Échantillonné par: Eva Roubeau	2025-01-30	Instantané
**Q25A046-05**	*1,3-DPG*		Eau	2025-02-07 08:00 EST<br>Échantillonné par: Eva Roubeau	2025-01-30	Instantané

† Les informations publiés dans cette section du certificat provient du client et sont trancrite du régistre de soumission.

RÉFÉRENCES

Méthode	Méthode d'analyse	Référence
	Q_O mykiss	SPE 1/RM/9

ESSAI DE LÉTALITÉ AIGUË SUR LA TRUITE ARC-EN-CIEL (CL50 - 96 HEURES) - RAPPORT

NUMÉRO DE LABORATOIRE: Q25A046-01

Analyste: VFL

DESCRIPTION DE L'ÉCHANTILLON:

Projet : DRCA / CMP-Elements critiques pour la technologie / 25-01
Échantillon testé: 6PPD

DESCRIPTION DES INSTALLATIONS ET DES CONDITIONS:

Les poissons ont été acclimatés à une température de 15 ± 2°C pendant au moins deux semaines avant d'être soumis à l'essai. Les poissons n'ont pas été nourris 16h avant l'essai. Les essais ont été réalisés dans des contenants de plastique revêtus de sacs jetables en polyéthylène. Les contenants d'essai ont été distribués de façon aléatoire. La hauteur des solutions d'essai était ≥ 15 cm. Durant l'essai, chaque contenant a été aéré au moyen d'air comprimé exempt d'huile passant par des pierres de barbotage propres (débit à 6,5 ±1 mL/min/L). La photopériode était de 16 ± 1 h de luminosité et 8 ± 1 h d'obscurité avec un éclairement de 100-500 lux à la surface des solutions d'essai. Les mesures de pH et de conductivité ont été prises à l'aide d'instruments effectuant une compensation automatique pour la température. Les mesures de conductivité sont rapportées à la température de référence de 25°C. Sauf indication contraire, toutes les conditions d'essai et les critères de validité spécifiés par les méthodes d'analyses d'Environnement et Changement climatique Canada ont été respectés.

Ajustement du pH: Aucun ajustement du pH.

PROTOCOLE UTILISÉ:

Essai réalisé selon les spécifications de la méthode d'Environnement et Changement climatique Canada, Méthode d'essai biologique: essai de létalité aiguë sur la truite arc-en-ciel, Méthode de référence SPE 1/RM/9, juillet 1990 avec modifications mai 1996 et mai 2007.

DÉVIATIONS À LA MÉTHODE:

Aucun élément inhabituel à propos du test.
Pour les besoins expérimentaux du chargé de projet des poissons pesant plus de 2,5g ont été utilisés pour l'exposition. De ce fait, la densité de chargement dépasse le seuil de 0,5 g/L.

ANALYSE REQUISE: CL50 - 96 heures (statique)

Volume par récipient: 60 L   Répétition: 1   Nombre de poissons / récipient: 10   Température d'essai (°C): 15 ± 1

EAU DE CONTRÔLE ET DE DILUTION: Eau douce (<10 ‰ v/v)   Source: Eau municipale traitée (déchlorée) Ville de Montréal

ORGANISME SOUMIS À L'ESSAI:

Espèce: Truite arc-en-ciel *Oncorhynchus mykiss*   Lot: 20241125LS   N° du réservoir d'acclimatation: 7

Source: Pisciculture LSL Living Seafoods Ltd. Hatchery   Pourcentage de mortalité 7 jours précédant l'essai: 0 %

POISSONS TÉMOINS: Statut des poissons témoins: aucune mortalité, comportement normal

Longueur à la fourche des poissons (cm)   Moyenne: 6,5   Minimum: 6,3   Maximum: 6,8

Poids frais des poissons (g)   Moyenne: 3,73   Minimum: 3,23   Maximum: 4,28   Densité de chargement (g/L): 0,6

CONDITIONS D'ESSAI - PARAMÈTRES PHYSICO-CHIMIQUES

Début de l'essai - date: 2025-01-30   Pré-aération débutée (h): 08:30   Durée de pré-aération (min): 30   Début de l'essai - heure: 09:00

Conc. (ug/L)		Témoin	Tém. DMSO	1,00	10,0	50,0	100	200
O.D. (%)	Initiale	101,0	101,7	101,2	101,5	100,9	101,1	100,9
	Finale	96,9	96,7	97,7	97,7	97,9	97,9	98,1
Temp. (°C)	Initiale	14,2	14,2	14,4	14,3	14,1	14,3	14,0
	Finale	14,5	14,3	14,4	14,6	14,4	14,8	14,5
pH	Initiale	8,12	8,13	8,10	8,12	8,13	8,12	8,14
	Finale	8,08	8,09	8,10	8,10	8,08	8,11	8,13
Conductivité (µS/cm)	Initiale	311	304	305	311	304	311	310

ESSAI DE LÉTALITÉ AIGUË SUR LA TRUITE ARC-EN-CIEL (CL50 - 96 HEURES) - RAPPORT

NUMÉRO DE LABORATOIRE: Q25A046-01

CONDITIONS D'ESSAI - MORTALITÉ CUMULATIVE / OBSERVATIONS †

	Concentration (ug/L)													
date/h des lectures	Témoin		Tém. DMSO		1,00		10,0		50,0		100		200	
	mortalité	obs.	mortalité	obs.	mortalité	obs.	mortalité	obs.	mortalité	obs.	mortalité	obs.	mortalité	obs.
2025-01-30 / 09:00	0	N	0	N	0	N	0	N	0	N	0	N	0	N
2025-01-31 / 09:10	0	N	0	N	0	N	0	N	0	N	0	N	0	N
2025-02-01 / 10:30	0	N	0	N	0	N	0	N	0	N	0	N	2	N
2025-02-02 / 09:50	0	N	0	N	0	N	0	N	0	N	0	N	2	N
2025-02-03 / 08:20	0	N	0	N	0	N	0	N	0	N	0	N	3	N

† **Observation:** termes décrivant l'aspect et le comportement des poissons (SPE 1/RM/9 juillet 1990 avec modifications de mai 1996 et mai 2007, Annexe E)

** Stress immédiat (Toux et nage erratique) ; + comportement ne peut être observé **Tégument:** ***A*** Décollé ***B*** Muqueux ***C*** Hémorragique ; **Pigmentation:** ***D*** Pâle ***E*** Foncée ***F*** Tachetée
Comportement général : ***G*** Passif ***H*** surexcitable ***I*** Irrité ***J*** En surface ***K*** En plongée ***L*** spasmodique ***M*** Tétanique ***N*** Normal **Δ** Moribond
Nage: ***O*** Interrompue ***P*** Irrrégulière ***Q*** Giratoire ***R*** Éclaboussante ***S*** Inversée ***T*** sur le flanc **Respiration:** ***U*** Rapide ***V*** lente ***W*** Toux ***X*** En surface ***Z*** Irrégulière

CONTRÔLE DE QUALITÉ: Toxique de référence

Date: 2025-01-24 CL50 - 96 heures (statique) = 0,28 mg/L Limites de confiance à 95%: 0,20 - 0,38

Produit chimique: Zinc Moyenne géométrique des CL50 = 0,40 mg/L Limites d'avertissement à 95%: 0,19 - 0,83 (n = 30)

RÉSULTATS D'ANALYSE:

Conc. (ug/L)	Témoin	Tém. DMSO	1,00	10,0	50,0	100	200
96 h Mortalité (%)	0	0	0	0	0	0	30

CL50 - 96 heures (statique) = >200 ug/L Limites de confiance à 95%: -

Où la concentration létale médiane (CL50) représente la concentration de matière dans l'eau, que l'on estime mortelle pour la moitié des organismes (50%) après une période d'exposition de 96 h.

Méthode statistique utilisée: aucun traitement

REMARQUES:

Composés : 6PPD
Fournisseur : LGC Toronto

Préparation solution d'essai
Solution stock (400 mg/L de 6PPD): 25,84 mg dans 65 mL de DMSO
Date de préparation: 2025-01-29 Analyste: Eva Roubeau Dumont

Volumes des contenants testés: 60 L

Concentrations (en ug/L)	Volume de la solution stock à ajouter (mL)
200	30
100	15
50	7,5
10	1,5
1	0,150

ESSAI DE LÉTALITÉ AIGUË SUR LA TRUITE ARC-EN-CIEL (CL50 - 96 HEURES) - RAPPORT

NUMÉRO DE LABORATOIRE: Q25A046-02 Analyste: VFL

DESCRIPTION DE L'ÉCHANTILLON:

Projet : DRCA / CMP-Elements critiques pour la technologie / 25-01
Échantillon testé: Mix Leachate

DESCRIPTION DES INSTALLATIONS ET DES CONDITIONS:

Les poissons ont été acclimatés à une température de 15 ± 2°C pendant au moins deux semaines avant d'être soumis à l'essai. Les poissons n'ont pas été nourris 16h avant l'essai. Les essais ont été réalisés dans des contenants de plastique revêtus de sacs jetables en polyéthylène. Les contenants d'essai ont été distribués de façon aléatoire. La hauteur des solutions d'essai était ≥ 15 cm. Durant l'essai, chaque contenant a été aéré au moyen d'air comprimé exempt d'huile passant par des pierres de barbotage propres (débit à 6,5 ±1 mL/min/L). La photopériode était de 16 ± 1 h de luminosité et 8 ± 1 h d'obscurité avec un éclairement de 100-500 lux à la surface des solutions d'essai. Les mesures de pH et de conductivité ont été prises à l'aide d'instruments effectuant une compensation automatique pour la température. Les mesures de conductivité sont rapportées à la température de référence de 25°C. Sauf indication contraire, toutes les conditions d'essai et les critères de validité spécifiés par les méthodes d'analyses d'Environnement et Changement climatique Canada ont été respectés.

Ajustement du pH: Aucun ajustement du pH.

PROTOCOLE UTILISÉ:

Essai réalisé selon les spécifications de la méthode d'Environnement et Changement climatique Canada, Méthode d'essai biologique: essai de létalité aiguë sur la truite arc-en-ciel, Méthode de référence SPE 1/RM/9, juillet 1990 avec modifications mai 1996 et mai 2007.

DÉVIATIONS À LA MÉTHODE:

Aucun élément inhabituel à propos du test.
Pour les besoins expérimentaux du chargé de projet des poissons pesant plus de 2,5g ont été utilisés pour l'exposition. De ce fait, la densité de chargement dépasse le seuil de 0,5 g/L.

ANALYSE REQUISE: CL50 - 96 heures (statique)

Volume par récipient: 60 L Répétition: 1 Nombre de poissons / récipient: 10 Température d'essai (°C): 15 ± 1

EAU DE CONTRÔLE ET DE DILUTION: Eau douce (<10 ‰ v/v) Source: Eau municipale traitée (déchlorée) Ville de Montréal

ORGANISME SOUMIS À L'ESSAI:

Espèce: Truite arc-en-ciel *Oncorhynchus mykiss* Lot: 20241125LS N° du réservoir d'acclimatation: 7

Source: Pisciculture LSL Living Seafoods Ltd. Hatchery Pourcentage de mortalité 7 jours précédant l'essai: 0,45 %

POISSONS TÉMOINS: Statut des poissons témoins: aucune mortalité, comportement normal

Longueur à la fourche des poissons (cm) Moyenne: 6,5 Minimum: 6,1 Maximum: 6,8

Poids frais des poissons (g) Moyenne: 3,55 Minimum: 3,04 Maximum: 4,22 Densité de chargement (g/L): 0,6

CONDITIONS D'ESSAI - PARAMÈTRES PHYSICO-CHIMIQUES

Début de l'essai - date: 2025-01-31 Pré-aération débutée (h): 08:05 Durée de pré-aération (min): 30 Début de l'essai - heure: 08:35

Conc. (%)		Témoin	0,001	0,01	0,1	0,3	3
O.D. (%)	Initiale	100,4	100,7	101,2	101,3	100,7	100,7
	Finale	97,6	98,1	97,7	97,6	97,5	97,8
Temp. (°C)	Initiale	13,9	14,4	14,1	14,0	14,2	14,1
	Finale	14,2	14,2	14,6	14,5	14,2	14,3
pH	Initiale	8,15	8,18	8,18	8,19	8,20	8,19
	Finale	8,07	8,10	8,08	8,09	8,09	8,09
Conductivité (μS/cm)	Initiale	306	308	306	306	308	301

ESSAI DE LÉTALITÉ AIGUË SUR LA TRUITE ARC-EN-CIEL (CL50 - 96 HEURES) - RAPPORT

NUMÉRO DE LABORATOIRE: Q25A046-02

CONDITIONS D'ESSAI - MORTALITÉ CUMULATIVE / OBSERVATIONS †

	Concentration (%)											
date/h des lectures	Témoin		0,001		0,01		0,1		0,3		3	
	mortalité	obs.	mortalité	obs.	mortalité	obs.	mortalité	obs.	mortalité	obs.	mortalité	obs.
2025-01-31 / 08:35	0	N	0	N	0	N	0	N	0	N	0	N
2025-02-01 / 10:10	0	N	0	N	0	N	0	N	0	N	0	N
2025-02-02 / 09:30	0	N	0	N	0	N	0	N	0	N	0	N
2025-02-03 / 07:55	0	N	0	N	0	N	0	N	0	N	0	N
2025-02-04 / 08:15	0	N	0	N	0	N	0	N	0	N	0	N

† **Observation:** termes décrivant l'aspect et le comportement des poissons (SPE 1/RM/9 juillet 1990 avec modifications de mai 1996 et mai 2007, Annexe E)
** Stress immédiat (Toux et nage erratique) ; + comportement ne peut être observé **Tégument:** ***A*** Décollé ***B*** Muqueux ***C*** Hémorragique **; Pigmentation:** ***D*** Pâle ***E*** Foncée ***F*** Tachetée
Comportement général : ***G*** Passif ***H*** surexcitable ***I*** Irrité ***J*** En surface ***K*** En plongée ***L*** spasmodique ***M*** Tétanique ***N*** Normal **Δ** Moribond
Nage: ***O*** Interrompue ***P*** Irrrégulière ***Q*** Giratoire ***R*** Éclaboussante ***S*** Inversée ***T*** sur le flanc **Respiration:** ***U*** Rapide ***V*** lente ***W*** Toux ***X*** En surface ***Z*** Irrégulière

CONTRÔLE DE QUALITÉ: Toxique de référence

Date: 2025-01-24 — CL50 - 96 heures (statique) = 0,28 mg/L — Limites de confiance à 95%: 0,20 - 0,38

Produit chimique: Zinc — Moyenne géométrique des CL50 = 0,40 mg/L — Limites d'avertissement à 95%: 0,19 - 0,83 (n = 30)

RÉSULTATS D'ANALYSE:

Conc. (%)	Témoin	0,001	0,01	0,1	0,3	3
96 h Mortalité (%)	0	0	0	0	0	0

CL50 - 96 heures (statique) = >3 % Limites de confiance à 95%: -

Où la concentration létale médiane (CL50) représente la concentration de matière dans l'eau, que l'on estime mortelle pour la moitié des organismes (50%) après une période d'exposition de 96 h.

Méthode statistique utilisée: aucun traitement

REMARQUES:

Composés : Mix Leachate
Solution stock 100% préparée par le groupe de la recherche

Volumes des contenants testés: 60 L

Concentrations (en %)	Volume de la solution stock à ajouter (mL)
3	1800
0,3	180
0,1	60
0,01	6
0,001	0,600

ESSAI DE LÉTALITÉ AIGUË SUR LA TRUITE ARC-EN-CIEL (CL50 - 96 HEURES) - RAPPORT

NUMÉRO DE LABORATOIRE: Q25A046-03

Analyste: VFL

DESCRIPTION DE L'ÉCHANTILLON:

Projet : DRCA / CMP-Elements critiques pour la technologie / 25-01
Échantillon testé: 6PPD-quinone

DESCRIPTION DES INSTALLATIONS ET DES CONDITIONS:

Les poissons ont été acclimatés à une température de 15 ± 2°C pendant au moins deux semaines avant d'être soumis à l'essai. Les poissons n'ont pas été nourris 16h avant l'essai. Les essais ont été réalisés dans des contenants de plastique revêtus de sacs jetables en polyéthylène. Les contenants d'essai ont été distribués de façon aléatoire. La hauteur des solutions d'essai était ≥ 15 cm. Durant l'essai, chaque contenant a été aéré au moyen d'air comprimé exempt d'huile passant par des pierres de barbotage propres (débit à 6,5 ±1 mL/min/L). La photopériode était de 16 ± 1 h de luminosité et 8 ± 1 h d'obscurité avec un éclairement de 100-500 lux à la surface des solutions d'essai. Les mesures de pH et de conductivité ont été prises à l'aide d'instruments effectuant une compensation automatique pour la température. Les mesures de conductivité sont rapportées à la température de référence de 25°C. Sauf indication contraire, toutes les conditions d'essai et les critères de validité spécifiés par les méthodes d'analyses d'Environnement et Changement climatique Canada ont été respectés.

Ajustement du pH: Aucun ajustement du pH.

PROTOCOLE UTILISÉ:

Essai réalisé selon les spécifications de la méthode d'Environnement et Changement climatique Canada, Méthode d'essai biologique: essai de létalité aiguë sur la truite arc-en-ciel, Méthode de référence SPE 1/RM/9, juillet 1990 avec modifications mai 1996 et mai 2007.

DÉVIATIONS À LA MÉTHODE:

Aucun élément inhabituel à propos du test.
Pour les besoins expérimentaux du chargé de projet des poissons pesant plus de 2,5g ont été utilisés pour l'exposition. De ce fait, la densité de chargement dépasse le seuil de 0,5 g/L.

ANALYSE REQUISE: CL50 - 96 heures (statique)

Volume par récipient: 60 L   Répétition: 1   Nombre de poissons / récipient: 10   Température d'essai (°C): 15 ± 1

EAU DE CONTRÔLE ET DE DILUTION: Eau douce (<10 ‰ v/v)   Source: Eau municipale traitée (déchlorée) Ville de Montréal

ORGANISME SOUMIS À L'ESSAI:

Espèce: Truite arc-en-ciel *Oncorhynchus mykiss*   Lot: 20241125LS   N° du réservoir d'acclimatation: 7

Source: Pisciculture LSL Living Seafoods Ltd. Hatchery   Pourcentage de mortalité 7 jours précédant l'essai: 0,57 %

POISSONS TÉMOINS: Statut des poissons témoins: aucune mortalité, comportement normal

Longueur à la fourche des poissons (cm)   Moyenne: 6,7   Minimum: 6,3   Maximum: 7,0

Poids frais des poissons (g)   Moyenne: 3,69   Minimum: 3,48   Maximum: 4,06   Densité de chargement (g/L): 0,6

CONDITIONS D'ESSAI - PARAMÈTRES PHYSICO-CHIMIQUES

Début de l'essai - date: 2025-02-06   Pré-aération débutée (h): 08:50   Durée de pré-aération (min): 30   Début de l'essai - heure: 09:20

Conc. (ug/L)		Témoin	Tém. DMSO	0,0050	0,050	0,50	5,0	50
O.D. (%)	Initiale	102,7	102,4	102,6	102,6	102,7	102,8	102,8
	Finale	97,5	97,8	97,7	97,9	97,0	99,2	99,5
Temp. (°C)	Initiale	14,5	14,6	14,5	14,5	14,3	14,7	14,7
	Finale	15,0	14,8	15,0	15,2	15,3	14,9	14,9
pH	Initiale	8,09	8,14	8,13	8,14	8,15	8,17	8,17
	Finale	8,01	8,02	8,01	8,01	8,00	8,13	8,12
Conductivité (µS/cm)	Initiale	306	309	306	306	307	309	309

ESSAI DE LÉTALITÉ AIGUË SUR LA TRUITE ARC-EN-CIEL (CL50 - 96 HEURES) - RAPPORT

NUMÉRO DE LABORATOIRE: Q25A046-03

CONDITIONS D'ESSAI - MORTALITÉ CUMULATIVE / OBSERVATIONS †

	Concentration (ug/L)													
date/h des lectures	Témoin		Tém. DMSO		0,0050		0,050		0,50		5,0		50	
	mortalité	obs.	mortalité	obs.	mortalité	obs.	mortalité	obs.	mortalité	obs.	mortalité	obs.	mortalité	obs.
2025-02-06 / 09:20	0	N	0	N	0	N	0	N	0	N	0	N	0	N
2025-02-07 / 09:20	0	N	0	N	0	N	0	N	0	N	8	N	9	N
2025-02-08 / 06:45	0	N	0	N	0	N	0	N	0	N	9	N	9	N
2025-02-09 / 09:45	0	N	0	N	0	N	0	N	0	N	9	N	9	N
2025-02-10 / 08:25	0	N	0	N	0	N	0	N	0	N	9	N	10	-

† **Observation:** termes décrivant l'aspect et le comportement des poissons (SPE 1/RM/9 juillet 1990 avec modifications de mai 1996 et mai 2007, Annexe E)

** Stress immédiat (Toux et nage erratique) ; + comportement ne peut être observé **Tégument:** ***A*** Décollé ***B*** Muqueux ***C*** Hémorragique **; Pigmentation:** ***D*** Pâle ***E*** Foncée ***F*** Tachetée
Comportement général : ***G*** Passif ***H*** surexcitable ***I*** Irrité ***J*** En surface ***K*** En plongée ***L*** spasmodique ***M*** Tétanique ***N*** Normal **Δ** Moribond
Nage: ***O*** Interrompue ***P*** Irrrégulière ***Q*** Giratoire ***R*** Éclaboussante ***S*** Inversée ***T*** sur le flanc **Respiration:** ***U*** Rapide ***V*** lente ***W*** Toux ***X*** En surface ***Z*** Irrégulière

CONTRÔLE DE QUALITÉ: Toxique de référence

Date: 2025-01-24 — CL50 - 96 heures (statique) = 0,28 mg/L — Limites de confiance à 95%: 0,20 - 0,38

Produit chimique: Zinc — Moyenne géométrique des CL50 = 0,40 mg/L — Limites d'avertissement à 95%: 0,19 - 0,83 (n = 30)

RÉSULTATS D'ANALYSE:

Conc. (ug/L)	Témoin	Tém. DMSO	0,0050	0,050	0,50	5,0	50
96 h Mortalité (%)	0	0	0	0	0	90	100

CL50 - 96 heures (statique) = 1,99 ug/L Limites de confiance à 95%: 1,29 - 3,08

Où la concentration létale médiane (CL50) représente la concentration de matière dans l'eau, que l'on estime mortelle pour la moitié des organismes (50%) après une période d'exposition de 96 h.

Méthode statistique utilisée: Spearman-Karber sans équeutage — Le logiciel CETIS (Tidepool Scientific Software) a été utilisé pour l'analyse des données.

REMARQUES:

Composés : 6PPD-quinone
Fournisseur : LGC Toronto — Pureté (%): 98,94

Préparation solution d'essai
Solution stock (100 mg/L de 6PPDQ): 5 mg dans 50 mL de DMSO
Date de préparation: 2025-02-05 — Analyste: Eva Roubeau Dumont

Volumes des contenants testés: 60 L

Concentrations (en ug/L)	Volume de la solution stock à ajouter (mL)
50	30
5,0	3,0
0,50	0,30
0,050	0,030
0,0050	0,003

ESSAI DE LÉTALITÉ AIGUË SUR LA TRUITE ARC-EN-CIEL (CL50 - 96 HEURES) - RAPPORT

NUMÉRO DE LABORATOIRE: Q25A046-04

Analyste: VFL

DESCRIPTION DE L'ÉCHANTILLON:

Projet : DRCA / CMP-Elements critiques pour la technologie / 25-01
Échantillon testé: HMMM

DESCRIPTION DES INSTALLATIONS ET DES CONDITIONS:

Les poissons ont été acclimatés à une température de 15 ± 2°C pendant au moins deux semaines avant d'être soumis à l'essai. Les poissons n'ont pas été nourris 16h avant l'essai. Les essais ont été réalisés dans des contenants de plastique revêtus de sacs jetables en polyéthylène. Les contenants d'essai ont été distribués de façon aléatoire. La hauteur des solutions d'essai était ≥ 15 cm. Durant l'essai, chaque contenant a été aéré au moyen d'air comprimé exempt d'huile passant par des pierres de barbotage propres (débit à 6,5 ±1 mL/min/L). La photopériode était de 16 ± 1 h de luminosité et 8 ± 1 h d'obscurité avec un éclairement de 100-500 lux à la surface des solutions d'essai. Les mesures de pH et de conductivité ont été prises à l'aide d'instruments effectuant une compensation automatique pour la température. Les mesures de conductivité sont rapportées à la température de référence de 25°C. Sauf indication contraire, toutes les conditions d'essai et les critères de validité spécifiés par les méthodes d'analyses d'Environnement et Changement climatique Canada ont été respectés.

Ajustement du pH: Aucun ajustement du pH.

PROTOCOLE UTILISÉ:

Essai réalisé selon les spécifications de la méthode d'Environnement et Changement climatique Canada, Méthode d'essai biologique: essai de létalité aiguë sur la truite arc-en-ciel, Méthode de référence SPE 1/RM/9, juillet 1990 avec modifications mai 1996 et mai 2007.

DÉVIATIONS À LA MÉTHODE:

Aucun élément inhabituel à propos du test.
Pour les besoins expérimentaux du chargé de projet des poissons pesant plus de 2,5g ont été utilisés pour l'exposition. De ce fait, la densité de chargement dépasse le seuil de 0,5 g/L.

ANALYSE REQUISE: CL50 - 96 heures (statique)

Volume par récipient: 60 L   Répétition: 1   Nombre de poissons / récipient: 10   Température d'essai (°C): 15 ± 1

EAU DE CONTRÔLE ET DE DILUTION: Eau douce (<10 ‰ v/v)   Source: Eau municipale traitée (déchlorée) Ville de Montréal

ORGANISME SOUMIS À L'ESSAI:

Espèce: Truite arc-en-ciel *Oncorhynchus mykiss*   Lot: 20250307LS_SPLIT(250417)   N° du réservoir d'acclimatation: 7

Source: Pisciculture LSL Living Seafoods Ltd.   Pourcentage de mortalité 7 jours précédant l'essai: 0 %

POISSONS TÉMOINS: Statut des poissons témoins: aucune mortalité, comportement normal

Longueur à la fourche des poissons (cm)   Moyenne: 6,5   Minimum: 6,2   Maximum: 7,0

Poids frais des poissons (g)   Moyenne: 3,65   Minimum: 3,34   Maximum: 3,90   Densité de chargement (g/L): 0,6

CONDITIONS D'ESSAI - PARAMÈTRES PHYSICO-CHIMIQUES

Début de l'essai - date: 2025-04-24   Pré-aération débutée (h): 08:25   Durée de pré-aération (min): 30   Début de l'essai - heure: 08:55

Conc. (mg/L)		Témoin	0,01	0,1	1
O.D. (%)	Initiale	100,5	100,9	101,5	101,3
	Finale	98,2	99,0	98,7	99,7
Temp. (°C)	Initiale	15,1	14,5	14,6	14,4
	Finale	14,0	14,7	14,3	14,2
pH	Initiale	8,18	8,14	8,13	8,12
	Finale	8,06	8,06	8,06	8,07
Conductivité (μS/cm)	Initiale	300	290	290	288

ESSAI DE LÉTALITÉ AIGUË SUR LA TRUITE ARC-EN-CIEL (CL50 - 96 HEURES) - RAPPORT

NUMÉRO DE LABORATOIRE: Q25A046-04

CONDITIONS D'ESSAI - MORTALITÉ CUMULATIVE / OBSERVATIONS †

	Concentration (mg/L)							
date/h des lectures	Témoin		0,01		0,1		1	
	mortalité	obs.	mortalité	obs.	mortalité	obs.	mortalité	obs.
2025-04-24 / 08:55	0	N	0	N	0	N	0	N
2025-04-25 / 08:10	0	N	0	N	0	N	0	N
2025-04-26 / 06:10	0	N	0	N	0	N	0	N
2025-04-27 / 14:20	0	N	0	N	0	N	0	N
2025-04-28 / 08:05	0	N	0	N	0	N	0	N

† **Observation:** termes décrivant l'aspect et le comportement des poissons (SPE 1/RM/9 juillet 1990 avec modifications de mai 1996 et mai 2007, Annexe E)

** Stress immédiat (Toux et nage erratique) ; + comportement ne peut être observé **Tégument:** ***A*** Décollé ***B*** Muqueux ***C*** Hémorragique **; Pigmentation:** ***D*** Pâle ***E*** Foncée ***F*** Tachetée
Comportement général : ***G*** Passif ***H*** surexcitable ***I*** Irrité ***J*** En surface ***K*** En plongée ***L*** spasmodique ***M*** Tétanique ***N*** Normal **Δ** Moribond
Nage: ***O*** Interrompue ***P*** Irrrégulière ***Q*** Giratoire ***R*** Éclaboussante ***S*** Inversée ***T*** sur le flanc **Respiration:** ***U*** Rapide ***V*** lente ***W*** Toux ***X*** En surface ***Z*** Irrégulière

CONTRÔLE DE QUALITÉ: Toxique de référence

Date: 2025-04-21 — CL50 - 96 heures (statique) = 0,29 mg/L — Limites de confiance à 95%: 0,22 - 0,38

Produit chimique: Zinc — Moyenne géométrique des CL50 = 0,37 mg/L — Limites d'avertissement à 95%: 0,17 - 0,81 (n = 30)

RÉSULTATS D'ANALYSE:

Conc. (mg/L)	Témoin	0,01	0,1	1
96 h Mortalité (%)	0	0	0	0

CL50 - 96 heures (statique) = >1 mg/L Limites de confiance à 95%: -

Où la concentration létale médiane (CL50) représente la concentration de matière dans l'eau, que l'on estime mortelle pour la moitié des organismes (50%) après une période d'exposition de 96 h.

Méthode statistique utilisée: aucun traitement

REMARQUES:

Composés : HMMM
Fournisseur : LGC Toronto — Pureté (%): 95

Préparation solution d'essai
Solution stock (100 mg/L de HMMM): 70mg dans 700mL d'eau distillée
Date de préparation: 2025-04-23 — Analyste: Eva Roubeau Dumont

Volumes des contenants testés: 60 L

Concentrations (en mg/L)	Volume de la solution stock à ajouter (mL)
1	600
0,1	60
0,01	6

ESSAI DE LÉTALITÉ AIGUË SUR LA TRUITE ARC-EN-CIEL (CL50 - 96 HEURES) - RAPPORT

NUMÉRO DE LABORATOIRE: Q25A046-05

Analyste: BW

DESCRIPTION DE L'ÉCHANTILLON:

Projet : DRCA/ CMP-Elements critiques pour la technologie/ 25-01
Description: 1,3-DPG, solution mère 8 g/L

DESCRIPTION DES INSTALLATIONS ET DES CONDITIONS:

Les poissons ont été acclimatés à une température de 15 ± 2°C pendant au moins deux semaines avant d'être soumis à l'essai. Les poissons n'ont pas été nourris 16h avant l'essai. Les essais ont été réalisés dans des contenants de plastique revêtus de sacs jetables en polyéthylène. Les contenants d'essai ont été distribués de façon aléatoire. La hauteur des solutions d'essai était ≥ 15 cm. Durant l'essai, chaque contenant a été aéré au moyen d'air comprimé exempt d'huile passant par des pierres de barbotage propres (débit à 6,5 ±1 mL/min/L). La photopériode était de 16 ± 1 h de luminosité et 8 ± 1 h d'obscurité avec un éclairement de 100-500 lux à la surface des solutions d'essai. Les mesures de pH et de conductivité ont été prises à l'aide d'instruments effectuant une compensation automatique pour la température. Les mesures de conductivité sont rapportées à la température de référence de 25°C. Sauf indication contraire, toutes les conditions d'essai et les critères de validité spécifiés par les méthodes d'analyses d'Environnement et Changement climatique Canada ont été respectés.

Ajustement du pH: Aucun ajustement du pH.

PROTOCOLE UTILISÉ:

Essai réalisé selon les spécifications de la méthode d'Environnement et Changement climatique Canada, Méthode d'essai biologique: essai de létalité aiguë sur la truite arc-en-ciel, Méthode de référence SPE 1/RM/9, juillet 1990 avec modifications mai 1996 et mai 2007.

DÉVIATIONS À LA MÉTHODE:

Aucun élément inhabituel à propos du test.
Pour les besoins expérimentaux du chargé de projet des poissons pesant plus de 2,5g ont été utilisés pour l'exposition. De ce fait, la densité de chargement dépasse le seuil de 0,5 g/L.

ANALYSE REQUISE: CL50 - 96 heures (statique)

Volume par récipient: 60 L   Répétition: 1   Nombre de poissons / récipient: 10   Température d'essai (°C): 15 ± 1

EAU DE CONTRÔLE ET DE DILUTION: Eau douce (<10 ‰ v/v)   Source: Eau municipale traitée (déchlorée) Ville de Montréal

ORGANISME SOUMIS À L'ESSAI:

Espèce: Truite arc-en-ciel *Oncorhynchus mykiss*   Lot: 20241125LS   N° du réservoir d'acclimatation: 7

Source: LSL Living Seafoods Ltd. Hatchery   Pourcentage de mortalité 7 jours précédant l'essai: 0 %

POISSONS TÉMOINS: Statut des poissons témoins: aucune mortalité, comportement normal

Longueur à la fourche des poissons (cm)   Moyenne: 6,8   Minimum: 6,7   Maximum: 7,0

Poids frais des poissons (g)   Moyenne: 4,05   Minimum: 3,72   Maximum: 4,40   Densité de chargement (g/L): 0,7

CONDITIONS D'ESSAI - PARAMÈTRES PHYSICO-CHIMIQUES

Début de l'essai - date: 2025-02-07   Pré-aération débutée (h): 08:05   Durée de pré-aération (min): 30   Début de l'essai - heure: 08:35

Conc. (mg/L)		Témoin	Tém DMSO	0,1	1
O.D. (%)	Initiale	102,7	102,4	102,3	101,9
	Finale	97,6	97,5	97,7	97,2
Temp. (°C)	Initiale	14,5	14,5	14,1	14,4
	Finale	15,0	15,2	14,8	15,0
pH	Initiale	8,16	8,17	8,19	8,22
	Finale	8,06	8,07	8,07	8,06
Conductivité (µS/cm)	Initiale	307	307	313	307

ESSAI DE LÉTALITÉ AIGUË SUR LA TRUITE ARC-EN-CIEL (CL50 - 96 HEURES) - RAPPORT

NUMÉRO DE LABORATOIRE: Q25A046-05

CONDITIONS D'ESSAI - MORTALITÉ CUMULATIVE / OBSERVATIONS †

date/h des lectures	Concentration (mg/L) Témoin		Tém DMSO		0,1		1	
	mortalité	obs.	mortalité	obs.	mortalité	obs.	mortalité	obs.
25-02-07 / 08:35	0	N	0	N	0	N	0	N
25-02-08 / 07:00	0	N	0	N	0	N	0	N
25-02-09 / 10:05	0	N	0	N	0	N	0	N
25-02-10 / 08:50	0	N	0	N	0	N	0	N
25-02-11 / 08:20	0	N	0	N	0	N	0	N

† **Observation:** termes décrivant l'aspect et le comportement des poissons (SPE 1/RM/9 juillet 1990 avec modifications de mai 1996 et mai 2007, Annexe E)
** Stress immédiat (Toux et nage erratique) ; + comportement ne peut être observé **Tégument:** ***A*** Décollé ***B*** Muqueux ***C*** Hémorragique **; Pigmentation:** ***D*** Pâle ***E*** Foncée ***F*** Tachetée
Comportement général : ***G*** Passif ***H*** surexcitable ***I*** Irrité ***J*** En surface ***K*** En plongée ***L*** spasmodique ***M*** Tétanique ***N*** Normal **Δ** Moribond
Nage: ***O*** Interrompue ***P*** Irrrégulière ***Q*** Giratoire ***R*** Éclaboussante ***S*** Inversée ***T*** sur le flanc **Respiration:** ***U*** Rapide ***V*** lente ***W*** Toux ***X*** En surface ***Z*** Irrégulière

CONTRÔLE DE QUALITÉ: Toxique de référence

Date: 2025-01-24 CL50 - 96 heures (statique) = 0,28 mg/L Limites de confiance à 95%: 0,20 - 0,38

Produit chimique: Zinc Moyenne géométrique des CL50 = 0,40 mg/L Limites d'avertissement à 95%: 0,19 - 0,83 (n = 30)

RÉSULTATS D'ANALYSE:

Conc. (mg/L)	Témoin	Tém DMSO	0,1	1
96 h Mortalité (%)	0	0	0	0

CL50 - 96 heures (statique) = > 100 mg/L Limites de confiance à 95%: -

Où la concentration létale médiane (CL50) représente la concentration de matière dans l'eau, que l'on estime mortelle pour la moitié des organismes (50%) après une période d'exposition de 96 h.

Méthode statistique utilisée: aucun traitement

REMARQUES:

Composés : 1,3-DPG
Fournisseur : LGC Toronto Pureté (%): n.d.

Préparation solution d'essai
Solution stock (8 g/L de 1,3-DPG): 160 mg dans 20 mL de DMSO
Date de préparation: 2025-02-05 Analyste: Eva Roubeau Dumont

Volumes des contenants testés: 60 L

Concentrations (en mg/L)	Volume de la solution stock à ajouter (mL)
1	7,5
0,1	0,75
